# The LIFE PERSUADED project approach on phthalates and bisphenol A biomonitoring in Italian mother-child pairs linking exposure and juvenile diseases

**DOI:** 10.1007/s11356-018-2660-4

**Published:** 2018-07-04

**Authors:** Cinzia La Rocca, Francesca Maranghi, Sabrina Tait, Roberta Tassinari, Francesca Baldi, Giorgia Bottaro, Emma Buzzigoli, Fabrizia Carli, Stefano Cianfarani, Raffaele Conte, Annalisa Deodati, Amalia Gastaldelli, Anna Paola Pala, Andrea Raffaelli, Chiara Saponaro, Giuseppe Scirè, Gian Luigi Spadoni, Luca Busani, the LIFE PERSUADED Project Group, Laura Narciso, Barbara Baldini Ferroli, Romana Marini, Daniela Germani, Graziano Barsotti, Demetrio Ciociaro, Veronica Della Latta, Graziella Distante, Melania Gaggini, Patrizia Landi, Antonio Di Virgilio, Andrea Martinelli, Mauro Valeri, Francesca Romana Mancini, Enrica Fabbrizi, Giacomo Toffol

**Affiliations:** 1 Center for Gender-Specific Medicine, Istituto Superiore di Sanità, Rome, Italy; 2 Department of Food Safety, Nutrition, Veterinary Public Health, Istituto Superiore di Sanità, Rome, Italy; 3 Dipartimento Pediatrico Universitario Ospedaliero “Bambino Gesu`” Children’s Hospital, Rome, Italy; 4 Tor Vergata University, Rome, Italy; 5 Institute of Clinical Physiology, CNR, Pisa, Italy, Pisa, Italy; 6 Department of Women’s and Children’s Health, Karolinska Institutet, Stockholm, Sweden; 7 Department of Infectious Diseases, Istituto Superiore di Sanità, Rome, Italy

**Keywords:** Human biomonitoring, BPA, DEHP, Children obesity, Puberty, In vivo toxicity

## Abstract

Phthalates and bisphenol A (BPA), plasticizers used in several products of daily life, are considered as endocrine disrupters, therefore children exposure is particularly relevant. The LIFE PERSUADED project aims to define the following: (a) the evaluation of internal levels of DEHP’s metabolites and BPA in Italian children and their mothers, (b) the association of the exposure with puberty development and obesity diseases, and (c) the effects of exposure in juvenile in vivo model. The cross-sectional study has involved 2160 mother-child pairs, including males and females, children and adolescents, from urban and rural areas of North, Center, and South Italy. A structured questionnaire and a food diary are designed to evaluate the association between lifestyle variables potentially related to DEHP/BPA exposure and internal levels, through univariate and multivariate analyses. Two pilot case-control studies are carried out on idiopathic premature thelarche and precocious puberty (30 girls each group, aged 2–7 years) and idiopathic obesity (30 boys and 30 girls, aged 6–10 years), matched to healthy controls. BPA and DEHP’s metabolites are analyzed in urine samples from all recruited subjects. Clinical and toxicological biomarkers are evaluated in serum of case-control subjects. Moreover, the toxicity study is carried out in a juvenile rodent model exposed to mixtures of BPA and DEHP at dose levels recorded in children population. The scientific results of LIFE PERSUADED will contribute to risk assessment of BPA and DEHP.

## Introduction

Phthalates and bisphenol A (BPA) are extensively used as plasticizers in several products of daily life. BPA is used in the production of plastic polycarbonate (PC) and epoxy-phenolic resins for non-food (paints, package surface coatings, printing inks, carbonless and thermal paper, tire manufacture, sealants used in dentistry, etc.) as well as for food applications (microwave ovenware, storage containers, refillable water containers, internal protective lining for food and beverage cans, etc.) (US Environmental Protection Agency 2010).

The main use of DEHP (di(2-ethylhexyl)phthalate) is in polymer products, mainly in flexible PVC for consumer and industrial uses such as toys, vinyl upholstery, car interiors, adhesives, and coatings. Moreover, DEHP is used in some food packaging and medical equipment (including injection bags and blood containers) (European Commission 2008).

Although DEHP and BPA are non-persistent chemicals, they are regularly detected both in the environment and in food (mainly in meats, fish, and dairy products (Serrano et al. 2014) due to their continuous release from manufacturing or processing facilities (Lorber et al. 2015); as a consequence, humans are exposed to both plasticizers, despite the restrictions (Covaci et al. 2015; Lee et al. 2014). Indeed, both DEHP and BPA are included in REACH Annex XVII restricted substances list; restrictions concern the manufacture of baby bottles containing BPA (Commission Directive 2011), although its use is permitted in food contact materials in EU, except France (Commission Regulation 2011), while DEHP use is restricted in toys and childcare articles (European Regulation 2007).

### BPA and DEHP effects on human health

BPA and DEHP act as endocrine disruptors (EDs) potentially affecting human health. They act on several organs: mainly the adipose tissue, reproductive organs, thyroid but also liver and pancreas.

Early studies demonstrated the presence in humans of BPA and phthalates and the associations with several reproductive and developmental disorders (Rochester 2013). BPA interacts with the activity of the estrogen pathways, but recently, it has been demonstrated to be involved in androgen and thyroid hormones signaling, causing adverse effect on sexual development and thyroid hormone homeostasis (Alonso-Magdalena et al. 2012). BPA also impacts the metabolic processes through “programming” effects evident later in life, like pancreatic insulin secretion (Soriano et al. 2012), possibly leading to type 2 diabetes and obesity onset (Alonso-Magdalena et al. 2011; Ben-Jonathan et al. 2009; Neel and Sargis 2011; Sun et al. 2014).

In vivo studies demonstrated that DEHP exposure alters the biosynthesis of estrogens and androgens, producing a significant decrease in serum testosterone levels as well as thyroid-disrupting effects. DEHP exposure is also implicated with alteration of lipid synthesis and adipogenesis since it activates the peroxisome proliferator-activated receptors (PPARs) and the Pregnane-X receptor (PXR) (Desvergne et al. 2009; Feige et al. 2007). PPARs are important for lipid metabolism and adipogenesis. Feige has shown that monoethyl-hexyl-phthalate (MEHP), a metabolite of DEHP, directly activates PPARg inducing the accumulation of lipids and enlargements of adipocytes (Feige et al. 2007). The same authors demonstrated that MEHP, but not DEHP, has an affinity for PPARg similar to rosiglitazone, a PPARg agonist.

The European Environment and Health Action Plan 2004–2010 (COM 2004) and, recently, EFSA (Choi et al. 2014) recognized the central role of human biomonitoring (HBM) to assess human exposure to chemicals, although it is not fully utilized yet. Limitations are mainly caused by lack of standardization in methodology (i.e., sample collection), validated biomarker availability as well as data integration and interpretation, as reported by EFSA supporting document (Choi et al. 2014). Several European initiatives, such as the DEMOCOPHES project (http://www.eu-hbm.info/democophes) in the past and HBM4EU at present (https://www.hbm4eu.eu/), have the objective to harmonize data and procedures in HMB studies throughout Europe and to establish protocols for the translation of HBM results into concrete policy recommendations.

EDs exposure during childhood deserves special attention since this represents a crucial susceptible phase of development. In particular, EDs seem to play a relevant role in precocious puberty and childhood obesity, two multi-factorial diseases whose incidence has increased in the last years. Recent studies have associated the exposure to DEHP and BPA in children to increased risk for endocrine-related diseases, thus posing the subjects at risk to develop type 2 diabetes and cardiovascular diseases early in life (Vafeiadi et al. 2017; Watkins et al. 2016). Indeed, phthalates exposure and urinary BPA levels have been positively associated with body mass index (BMI) or waist circumference (WC) respectively in Chinese school children (Wang et al. 2013) and in American children in the CHAMACOS cohort study (Harley et al. 2013).

In Italy, the prevalence of childhood obesity varies from 5% in the North up to 20% in the South. Early signs of puberty have been reported in more than 6% of Italian girls (Russo et al. 2012). A high incidence of central precocious puberty in a circumscribed industrial area in North West Tuscany was reported and ascribed to exposure to undefined EDs (Massart et al. 2005). No national data exist on levels of DEHP and its metabolites (MEHP and secondary metabolites) and/or BPA in toddlers, children, and adolescents or associations between exposure to these compounds and early puberty or obesity.

The LIFE PERSUADED project (“Phthalates and bisphenol A biomonitoring in Italian mother-child pairs: link between exposure and juvenile diseases”), funded by the European Commission (under grant agreement LIFE13 ENV/IT/000482), aims to (a) estimate the exposure of Italian children and their mothers to DEHP metabolites and BPA through a human biomonitoring study also taking into account geographical and demographic differences; (b) link environmental exposure to children health, through children case-control studies on idiopathic premature thelarche, idiopathic central precocious puberty and children idiopathic obesity also integrating clinical and biomarkers of effect levels as proxy of potential adverse outcomes; (c) evaluate cause-effects due to BPA/DEHP exposure in experimental conditions by a juvenile toxicity study in rodents, as innovative animal model for childhood diseases.

The project started at the end of 2014 and it will be concluded at the end of 2018; at present, we have completed the enrollment of the subjects and the animal treatments, whereas the analyses of samples and data evaluation are still ongoing. This paper aims to illustrate the innovative and integrated approach of the project and to provide methodological reference for other scientists interested in HBM studies and integrated approaches for the risk assessment of environmental contaminants.

## Methods and analysis

The LIFE PERSUADED approach has three main objectives:

- Objective 1—Human biomonitoring study (HBM)

The main goal of LIFE PERSUADED is to determine internal levels of BPA, DEHP metabolites in mother-child pairs from urban and rural areas of three Italian geographical macro-areas (North, Center, South), as different scenarios of exposure, to set background levels for two relevant susceptible groups of population. Moreover, the HBM results will provide contribution on risk assessment by developing biomonitoring equivalents and HBM1 and HMB2 reference values for BPA and DEHP in children and in women, and definition of a panel of biomarkers specifically correlated to BPA/DEHP human exposure and health status.

- Objective 2—Case-control studies

To assess the risks associated with EDs exposure, the project investigates the association between the exposure to DEHP/MEHP and/or BPA and idiopathic dysregulations of pubertal development, such as premature thelarche, precocious puberty, and juvenile idiopathic obesity in the Italian young population.

- Objective 3—Toxicological study

A targeted in vivo experimental study is performed on rodents at juvenile life stage (from weaning to sexual maturity). The juvenile sub-acute toxicity study is designed to evaluate the potential modes of actions and specific effects of BPA, DEHP and mixtures of them, mimicking the real exposure scenario for children population. The dose levels for the animals were defined tacking into account the preliminary results of the biomonitoring study, in order to represent the actual exposure of the Italian children and adolescents.

### Human biomonitoring study design (HBM)

The cross-sectional study involves 2160 mother-child pairs, including males and females, children and adolescents, splitted into three age groups: 4–6, 7–10, and 11–14 years. The sampling was designed to consider age groups, gender, demographical area (urban and rural), and geographical macro-area (North, Center, and South Italy). The sampling size was defined to estimate the internal levels of BPA and phthalates and to evaluate any difference among each “strata,” considering as a reference the values obtained from the DEMOCOPHES HBM study (http://www.eu-hbm.info/democophes). Thus, 60 healthy mother-child pairs from each “stratum” (age, gender, and demographical and geographical areas) was estimated to be enrolled.

For the enrollment of children and mothers, the following inclusion/exclusion criteria have been considered: residence in the selected area for at least 6 months prior to the enrollment, healthy status, children body mass index between 5th and 85th percentile, and age between 4 and 14 years.

Children with current diseases, diarrheal episodes within the 5 days before urine sampling or suffering from chronical diseases were excluded, as well as mothers in gestational status or in breastfeeding. Each enrolled mother-child pair was asked to deliver a sample of urine, sign the informed consent and fill in the questionnaire and food diary related to the 2 days before sampling. All samples were shipped to IFC-CNR for analysis. An aliquot of collected urine sample will be stored in the IFC-CNR biobank (https://www.ifc.cnr.it/index.php/it/le-aree-di-competenza/la-biomedicina/bio-banca) to create a human specimens’ repository useful in future research on similar topic.

#### Geographical areas selection

The Italian territory was subdivided into three macro-areas: North, Center, and South. Within each macro-area, urban and rural areas were defined based on population density (> or < 150 inhabitants/Kmsq) and number of inhabitants (> or < 50.000 inhabitants) according to the data provided by the Italian Institute of Statistics (ISTAT).

#### Network of pediatricians

The enrollment of children and mothers in the HBM study was carried out by Family Pediatricians of the Italian National Health System (NHS). Two networks of family pediatricians were involved, namely, the *“*Associazione Culturale Pediatri” (ACP, http://www.acp.it/) and “Medici Pediatri Marche” (FIMP Marche, http://www.fimpmarche.it/). A total of 86 pediatricians of 11 Italian regions (3 in the North, 3 in the Center, and 5 in the South macro-areas) agreed to participate to the project and a network of pediatricians has been set up for training events organization and recruitment. For each region, a responsible was appointed attending specific training sessions at the ISS. In turn, the representative pediatricians trained and coordinated the colleagues from the same region, under the supervision of the project coordinator. During the training events, the pediatricians were informed about the design of the study, inclusion/exclusion criteria to be applied for the enrollment of mother-child pairs, management of tools required by the study, i.e., informed consent, questionnaire, and food diary available in both paper and online formats, and kit for urine sampling. The pediatricians, during their routine activity, were requested to select eligible candidates as mother-child pairs, to present them the study, ask them to sign the informed consent, explain how to fill in the questionnaire/food diary, and collect urine samples for analytical determination of BPA and DEHP metabolites levels.

### Case-control studies

Three pilot case-control studies were carried out on idiopathic premature thelarche (IPT) and precocious puberty (IPP, a minimum of 30 girls each group, aged 2–7 years) and idiopathic obesity (IO, a minimum of 30 boys and 30 girls, aged 6–10 years). The enrollment of the subjects was performed at the “Bambino Gesù” Children’s Hospital, according to the following inclusion criteria for each specific pathology:

- For IPT: age 2–7 years; mono or bilateral breast development (Tanner stage II); absence of other signs of puberty; normal stature and growth rate; bone age corresponding to chronological age or advanced of no more than 1 year; pelvic ultrasound scan consistent with pre-puberty: uterine longitudinal length and volume < 3.5 cm and < 1.8 ml respectively, ovarian volume < 3 ml, no ovarian follicles with a diameter > 9 mm; pre-pubertal estradiol levels (< 20 pg/ml); pre-pubertal GnRH test (peak FSH value higher than LH, peak LH value < 5 mIU/ml); and no concomitant therapies
- For IPP, age 2–7 years; mono or bilateral breast development (Tanner stage II); accelerated growth rate (≥ 75th centile, according to Tanner standards); advanced bone age (at least more than 1 year, according to Greulich and Pyle method); pubertal pelvic ultrasound scan: uterine length > 3.5 cm and volume > 1.8 ml; pubertal estradiol (> 20 pg/ml); pubertal GnRH test (peak LH value > 5 mIU /ml); brain MRI scan negative for alterations; no concomitant therapies; and no family history for precocious puberty
- For IO, age 6–10 years; BMI > 90th; no endocrine and genetic disorders; and no concomitant therapies
- Healthy controls matched 1:1 by age and gender related to each pathology were also recruited

Urine and serum samples were collected from all the subjects for BPA and DEHP metabolites’ analyses, and for serum biomarkers assessment, respectively.

### Biomarkers of effects

HBM approach in LIFE PERSUADED aims to integrate internal EDs levels, as biomarker of exposure, with biomarkers of health effects, derived from clinical use as well as from toxicological evidence, proxy of potential adverse effects. The use of biomarkers of effect as indicator of association between internal exposure and infertility has been adopted in a previous study concerning BPA, DEHP and PFC human exposure (La Rocca et al. 2014; La Rocca et al. 2015).

For the subjects enrolled in the case-control studies, the results of the routinely clinical and biochemical analyses are collected in a clinical dataset, namely, markers of glucose metabolism (oral glucose tolerance test, C peptide, HbA1c, IGF1, and IGFBP3), lipid profile (total cholesterol level, triglycerides level, HDL, and LDL), hepatic function (AST, ALT, and GGT), and calcium metabolism (vitamin D, Parathyroid hormone, calcium, Phosphorus, and Alkaline phosphatase).

Besides, for each subject of the case-control studies, four additional biomarkers of effect, namely, leptin, adiponectin, anti-Mullerian hormone (AMH), and kisspeptin, are assessed as crucially involved in reproductive and metabolic pathways. Leptin and adiponectin are involved in the regulation of glucose and lipid metabolism, food intake, energy expenditure, and inflammatory response (Lee and Shao 2014; Makki et al. 2013; Stern et al. 2016).

Leptin plays also a fundamental role in the regulation of menstrual cycle triggering kisspeptin production, which, in turn, induces both LH and FSH (Sanchez-Garrido and Tena-Sempere 2013). Kisspeptin has been recently demonstrated to participate in the regulation of the gonadotropin-releasing hormone (GnRH) at puberty. Since the activation of GnRH is a prerequisite to puberty onset, a decrease in kisspeptin levels at this developmental stage may severely affect puberty progression (Abaci et al. 2015; Pinilla et al. 2012). AMH, regulating the folliculogenesis, is proposed as a novel marker of ovary function, in addition to canonical hormones in normal peripubertal girls (Dayal et al. 2014; Dewailly et al. 2014). The selected biomarkers are assessed by commercial ELISA kits.

### Questionnaire and food diary

A structured questionnaire and a food diary were designed to evaluate the association between demographic and lifestyle variables potentially related to DEHP/BPA exposure and internal levels in children and their mothers. The former aims collecting information on residential environment, nutrition, smoking behavior, socio-demographic data, lifestyle, and food habits of subjects, also taking into account the output of DEMOCOPHES project on HBM.

The food diary was developed to gather detailed information about food items consumed 2 days before the urine sampling, adding specific questions concerning the handling, cooking, and storage of the foods consumed, considering any potential source of BPA and phthalates measured in the urine samples.

After a pre-test on about ten mothers, the final version of the questionnaire and the food diary was edited in paper format as well as put in electronic format by the IFC-CNR group which implemented the electronic version on a self-hosted server (http://questionario-persuaded.ifc.cnr.it/). The enrolled subjects in HBM and case-controls studies are asked to fill in the questionnaire and the food diary.

### Data management and quality assessment

A coding system to assign unique identifier IDs to the recruited subjects has been established, and at the recruitment, assigned to each mother-child pair. These IDs were created also to accomplish the Ethics Committee requirements concerning data protection and anonymity. The IDs are alphanumerical codes (five numbers and one letter) identifying the pediatrician, the mother-child pair and distinguishing between mother (W) and child (C; i.e., 100-01 C and W). Each code, assigned to subjects enrolled in the HBM and in the case-control studies, has to be attached on the corresponding questionnaire/food diary and on the disposable vessels for urine sampling. The same code is used to access the online questionnaire and food diary since the access is restricted to the study participants and the coordinators of the institutions involved in the data collection and management.

A relational database (MySQL) to manage all the data was developed and hosted on virtualized environment of the IFC-CNR IT infrastructure.

A quality control of the data entry on both questionnaires and diaries was developed to ensure the completeness and the quality of data. The questionnaire and the food diary are self-explaining, allowing no blank answer during the filling in.

The enrolled subjects may choose to fill in the questionnaire and food diary using the paper or the electronic version. In the first case, the data entry will be performed by the IFC or ISS staff members.

Any incoherent entry is identified and evaluated by the operators in charge of data quality control and, if the case, clarification is asked to the pediatrician who enrolled the subject and supervised the data and sampling collection. A dedicated help-desk managed by a trained people was also considered to (i) ensure harmonization of the procedures of data entry and quality assessment, (ii) manage any related problem, and (iii) support pediatricians and the people in the different institutions in charge of the data collection and entry.

### In vivo experimental study

Male and female juvenile rats were exposed orally by gavage for 28 days—from Post Natal Day (PND) 23 corresponding to weaning until sexual maturity—with three dose levels each of BPA, DEHP and their mixtures; the dose levels were derived from levels of contaminants detected in children population (*N* = 10/sex/group). BPA dose levels were obtained by transforming detected children population internal levels in estimated intake from creatinine-based urinary concentration, according to EFSA recommendations (EFSA and CEF Panel 2015). The same calculation was applied to DEHP, considering that the 99% of DEHP is converted in MEHP. Calculated dose levels are between 2 and 18 mg/kg body weight (bw) per day for BPA and between 9 and 48 mg/kg bw per day for DEHP. The treated animals are compared with a group of control for each gender. Parameters of sexual development for each sex (timing of vaginal opening and preputial separation) were recorded during the treatment period. At sacrifice, all the rats, including controls, were anesthetized with a gaseous solution of isofluorane; blood samples were collected by intracardiac puncture for the determination of 17-β estradiol, testosterone, T3, TSH, leptin, adiponectin, kisspeptin, and anti-Mullerian hormone serum levels. Subsequently, animals were sacrificed by CO_2_ asphyxiation. At sacrifice, spleen, uteri, ovaries, testes, liver, pancreas, thyroid, and adrenals were excised, weighed, and stored for histomorphological and histopathological analyses. Tissues were embedded in paraffin, cut into 5-μm sections, and stained with hematoxylin/eosin and PAS method for the examination under a light microscope. Quantitative histomorphometrical analyses will be performed according to Maranghi et al. (2009) on spleen, uterus, ovaries, thyroid, and adrenals. Briefly, slides of tissues will be examined by means of an image analysis system applied to the light microscope. Gene expression at hypothalamic level will be performed. Briefly, hypothalami were flash frozen in liquid nitrogen and stored at − 80 °C; total RNA will be extracted by a commercial kit and analyzed for thyroid-stimulating hormone (TSH), luteinizing hormone (LH), and follicle stimulating hormone (FSH) genes expression by real-time PCR.

### BPA and DEHP metabolites assessment

BPA and DEHP metabolites (MEHP, 5OH-MEHP, and 5oxo-MEHP) levels are determined on urine samples in subjects from HBM and case-control studies. Upon arrival to CNR laboratory, samples are checked, catalogued, and aliquoted for sample analysis and storage in the biobank. As phthalates and BPA are metabolized and excreted in the urine in a glucuronidated form (that is soluble in water, while phthalates and BPA are hydrophobic); the analysis requires a previous enzymatic deconjugation (β-glucuronidase enzyme from *Helix pomatia*, ≥ 100.000 units/mL Sigma Aldrich) to hydrolyze the glucuronide form and separate free phthalate metabolites and BPA. Samples are purified through individual SPE cartridge C18 ODS 3 ml tubes 200 mg (Agilent), and then analyzed by LC–ESI-MS QTOF (Agilent UHPLC 1290 infinity coupled with Agilent 6540 Quadrupole Time-of-Flight (QTOF) system equip with Agilent ZORBAX SB-Phenyl 2.1x100mm 1.8-Micron), for DEHP’s metabolites and by GC/MS (Agilent GC-7890 coupled with Agilent MS-5975C) for BPA quantification. Since samples are from spot urine, they need to be normalized with respect to creatinine concentration. For each subject, creatinine concentrations were measured by AU400 Beckman analyzer using an enzymatic method (Beckman Coulter).

### Data analysis plan

A data analysis plan to define final criteria for inclusion in the analysis, data management, and statistical analyses has been developed. The software STATA 14.0 (StataCorp, Texas, TX, USA) will be used.

As a first step, the final dataset will be set up excluding subjects: (i) who do not accomplish to the inclusion criteria established in the enrollment protocols for the HBM and case-controls studies (age, sex, and BMI); (ii) for which the urinary concentrations of phthalates and BPA are not available (urine samples not available, sample lost or broken during transfer to central lab, or are not eligible to be analyzed); (iii) for which urinary analytical results are qualitatively not acceptable (results not consistent with chemical quality analysis standards).

The subjects with more than 80% of missing values in questionnaire and food diary are included only in the estimate of the internal levels of DEHP’s metabolites and BPA. According to the ethic committee requirements for data protection and anonymity, all the data will be presented aggregated.

Descriptive analyses: for quantitative variables, the measures of central tendency (mean, median, and mode) and dispersion (standard deviation and percentiles) will be calculated. For qualitative variables, the frequency and the confidence intervals according to the binomial distribution will be calculated.

The correlation between the outcome variables (internal exposure level of phthalates and BPA) and the other predictive variables will be performed in univariate and multivariate analyses. Qualitative (dicotomous) variables will be created according to the percentiles (over 50%, over 75%, and over 90%) from the internal exposure level of phthalates and BPA and the correlation with qualitative predictors will be analyzed. Other qualitative variables will be created by aggregating different variables to define specific “profiles” in particular for the aggregated analysis of lifestyles, behaviors, food consumption, and food handling.

### Ethics committee approval

Since the core project activity concerns collection of information and samples from children and their mothers, the evaluation of the ethic aspects by Ethics Committee was a basic requirement. Ethics Committees of the Istituto Superiore di Sanità (National Health Institute), as project coordinator, and “Bambino Gesù” Children Hospital, as beneficiary in charge of the case-control studies, approved the project and the informed consent. The approved consent administered to mothers and children (enrolled in both HBM and case-control studies), ensured data protection and anonymity, described management of information and samples, study design, and statistical power.

With respect to the animal study, the protocol for the animal experiment was approved by the internal OPBA (Organo Preposto al Benessere Animale – Animal Welfare Group at ISS) and subsequently by the Italian Ministry of Health. All the animal experiments are conducted in compliance with the European Council Directive 86/609/EEC and the more recent 2010/63 (European Directive 2010) as well as the Italian Legislation on Animal Experimentation (D. Lvo 26/14). The Directive and the Italian law by themselves guarantee the application of 3Rs (OECD Principles on Good Laboratory Practice).

### Communication and dissemination activity

Communication is a relevant activity throughout the project, from the recruitment to the results dissemination; all initiatives, news, events, publications, and activities related to the project are available on the project website (https://lifp.iss.it/) core element of the external communication and dissemination plan of the project.

Periodically, a newsletter issue focused on LIFE PERSUADED subjects is published online and sent to a mailing list of scientists and NGO associations.

LIFE PERSUADED approaches are disseminated to several target audiences, i.e., scientists, NGO, stakeholders, doctors’ associations, and general public, by adopting different strategies and appropriate languages. Indeed, at the beginning of the project, notice boards were produced for scientists and for the general public. Particular attention is dedicated to dissemination to the general population: leaflets were printed out and distributed with the objective to inform about the aims and contents of the project and to promote the recruitment of mother-child pairs and case-control studies (leaflet for recruitment); likewise, at the end of the project, “leaflet for feedback” will be drown up to communicate the results to enrolled people. Social media, such as Twitter and Facebook, is also used to disseminate relevant information, especially to an adolescent audience.

General public, NGOs, as well as scientists and policy-makers, will be also the target of workshops, organized by the project to disseminate preliminary and final results to discuss the relevance of and interest in actions at national and EU level. Project results may contribute as well to promote the consciousness and awareness of people on exposure to EDs to adopt healthier lifestyles, avoiding or reducing the use of products as potential source of exposure.

The first event held in December 2015 allowed to get in touch with stakeholders and to set the stage for the future networking action among LIFE projects.

## Results

The scientific results of PERSUADED will provide a baseline for future risk assessment of BPA and DEHP, with new data concerning the following: (i) the exposure of children and their mothers in Italy, (ii) the toxicity endpoints emerged from animal models, and (iii) the association with some endocrine-related diseases in children from the case-control studies. The human samples biobank developed during the project will be maintained and made available for other researches in similar field, upon request. The communication and dissemination activity, tools, and material will contribute to increase the awareness of the institutions and the public concerning EDs. The national network of family pediatricians established within the project is valuable and will facilitate future collaboration to promote mother-children health, as requested by the national policies on dismetabolic diseases in childhood. LIFE PERSUADED will support the formulation and implementation of community policies to reduce the public health impact of chemicals, with emphasis on the health risks in the early stages of life; besides that, LIFE PERSUADED also addresses the growing consumer concerns with regard to chemical and toxicological risks related to food and environment.
